# Student writing in the engineering curriculum: discursive rhetorical model of the laboratory report genre in Spanish

**DOI:** 10.3389/fpsyg.2023.1303280

**Published:** 2023-11-15

**Authors:** Enrique Sologuren

**Affiliations:** Instituto de Literatura, Universidad de Los Andes, Las Condes, Chile

**Keywords:** student genre, macro-genre, mesogenre, lab report genre, curriculum, civil engineering computer science

## Abstract

**Introduction:**

The laboratory report is a widely used genre in the academic training process in civil computer engineering. Students produce this genre in the university classroom for diverse academic and professional purposes. Despite its relevance, empirical rhetorical-discursive descriptions of the value of student writing are still scarce in Spanish. Thus, we describe the rhetorical-discursive organization of the laboratory report genre in this subdiscipline.

**Methods:**

To fulfil this purpose, we followed a methodological design based on Swalesian Genre Analysis and used a corpus of ninety-eight texts. The sample was collected in a self-compiled form through consultation with teachers and students in the university classroom. The application of this method allowed us to determine the macro-moves, moves, and rhetorical steps of the genre, its communicative functions, and textual features.

**Results:**

The resulting rhetorical model consisted of four macro-moves, twelve moves and seventeen steps. This model shows the highly dynamic and mesogeneric nature of this genre, the new functions of multimodal artefacts, and the genre’s presence across the curriculum. To know about the teachers’ and students’ views on the process of training professional writers in engineering, the rhetorical model was complemented with an ethnographic phase (in the terms proposed by Swales: corroboration process with a couple of members of the community) before and after collecting the textual corpus.

**Discussion:**

Finally, the implications for genre theory, Spanish language theory and pedagogy of the Spanish language and genre pedagogy are discussed.

## 1 Introduction

Research on university students as writers in the disciplines is still a vast area of exploration. Despite the advances at an international and national level (Calle-Arango and Avila-Reyes, 2020), new descriptions and reflections must be incorporated that connect the discursive production with the broader educational and professional practices. Thus, the production of laboratory reports stands out within the academic genres used in undergraduate education. The laboratory report (in Spanish, Informe de Laboratorio, and from now on called ILAB) is a hybrid academic training genre characteristic of curricular and scientific writing. This type of genre can be identified in different computer engineering courses, ranging from the initial cycle to the professional or terminal cycle. The methodology of working in a laboratory is common in scientific education and professional training; however, its mediation through texts has been scarcely explored. ILAB is a commonly used genre in the teaching and learning processes in the area of engineering and sciences as a suitable medium to report findings of empirical studies (Parkinson, 2017). Moreover, Lerner (2007) traces interest in the teaching of this genre dating from at least 1890. This interest continues and the laboratory’s teaching and learning processes are an object of study in the scientific education of multiple disciplines, mostly due to the impact that both the traditional method and more recent inquiry-based (Resendes, 2015) and virtual simulation approaches (Rodríguez-Llerena and Llovera-González, 2014) have on learning.

Furthermore, recently this genre has been identified and described in other disciplinary areas such as psycholinguistics and computational linguistics (Dreyfus et al., 2016) and biology in first and second year at university (Humphrey and Hao, 2013; Dreyfus et al., 2016), in the context of undergraduate academic training at an Australian university. In this vein, Martin and Rose (2008) identify similar genres in the science field in the secondary classroom, and they classify them within the genre family of “procedural recounts” of great relevance for scientist-researchers. This family includes technical notes, research articles, and experiment reports. This genre also aligns with what are called the school science genres (Veel, 1997) in which students complete the experiments in a laboratory and then present the results. In this sense, we can conclude that the Laboratory Report is an academic training genre in civil engineering (GEFIC) with an applied orientation or implementation genre (Author). In Latin America, studies on academic genres and genres “report” in general stand out (Merodo and Natale, 2012; Navarro and Chiodi, 2013; González and Burdiles, 2018; Meza and Da Cunha, 2019; Londoño-Vásquez and Ramírez Botero, 2020).

Our research problem seeks to answer the question *How are communicative purposes organized in a training genre highly relevant to civil engineering education?* The objective is to describe the rhetorical-discursive organization of the laboratory report genre in this subdiscipline due to their theoretical and applied impact on the training of advanced writers. This article structure is as follows: we begin with a theoretical conceptualization and a literature review of the report as a genre and the technical report as a macro-genre. Then we develop the research methodology, followed by the results, which include the rhetorical-discursive model developed. Finally, we provide a discussion and conclusions on genre description in the Spanish language and the training of writers in the 21st century’s multimodal society.

## 2 State of the art

### 2.1 The report as an academic training genre

Academic writing is a key activity for secondary and higher education students. Although the specialized literature on academic training genres is still incipient, it is possible to trace approaches to academic writing of students at different education levels; thus, some studies have explored argumentative textual sequences (Hael, 2012; López, 2012; Fernández et al., 2017), explanatory (Albano de Vázquez et al., 2001) and descriptive ones (Oyanedel, 2005). The report genre has received greater attention from specialists in the Latin American settings, who have described some thematic and figures of speech construction mechanisms (Oyanedel, 2006), lexical patterns associated with disciplinary identities (Muñoz, 2006; López-Bonilla, 2013), and students’ difficulties for information processing to be able to generate texts with an intention, texts that are autonomous, and texts that construct and reconstruct disciplinary knowledge (Tapia Ladino et al., 2003; Vázquez and Miras, 2004; Tapia Ladino and Burdiles, 2009; Navarro, 2014; Trigo Ibáñez and Núñez-Sabaris, 2018).

The academic report genre has been defined as an interaction fulfilling a particular social purpose within the various discursive practices used by students in undergraduate programs, which makes it possible to estimate a level of development in a specific disciplinary area (Núñez and Espejo, 2005). In an academic report, both a university student as the utterer and an academic as the receiver, who assesses the application of textual-discursive applications (Harvey and Muñoz, 2006; Núñez et al., 2006). The report genre has several names depending on the academic communities, and it is recognized as a relevant textual artifact in undergraduate education (Harvey and Muñoz, 2006).

Harvey’s (2005) analysis distinguishes between four types of reports: bibliographical, diagnostic, case, and research report. On the other hand, Tapia Ladino and Burdiles (2009) analyzed a corpus of 208 reports produced in twenty-eight programs (3° and 4° year) of four majors representing different areas: health, sciences, education, and economy. They found six types of reports: case, research-article-like, questionnaire, monograph, observation, and teaching intervention proposal. The case report exhibited the highest internal variability, while on the other extreme is the teaching intervention proposal. This characterization and distribution of the texts that are actually produced by students leads the researchers to reflect on this genre’s functionality:

Distinguishing between texts that favor the learning processes within the classroom from those that are addressed to the lecturer as a member of the discipline or the profession can shed light on the demands our students are subject to Tapia Ladino and Burdiles (2009, p. 43).

Moreover, recent studies in the Anglo-Saxon context have looked into this genre and its linguistic and textual configuration. Gardner (2012) develops a register analysis and compares the structural organization of student-written reports belonging to two generic families: the research report and the methodology report. A linguistic and discursive description of the laboratory report has been proposed by Parkinson (2017), who points out that these types of discourses have been studied by the scientific education and the teaching of scientific writing as learning and assessment instruments, but not from a linguistic and discursive point of view.

Even though the report has been the most frequently studied academic-student training genre, studies have recently emerged that are focused other writing practices of the academic discourse, such as the academic-teaching genre essay-type test (Farlora, 2015), which is defined as a projection of general school genres (Parodi, 2008) and “as part of an academic-evaluative macro-genre whose purpose is to assess the degree of belonging to a disciplinary community” (Farlora, 2015, p. 263). A set of research proposals in the literature analyze strategies to introduce university students to the knowledge and use of expert genres such as the conference presentation (Padilla and Carlino, 2010), the review (Alzari et al., 2014), and case study (Merodo and Natale, 2012; Ávila, 2016).

Therefore, more information is needed about *what* students write during the different stages in their academic training and various academic literacy levels (Marinkovich and Córdova, 2014; Marinkovich and Poblete, 2014). Moreover, more studies based on broad text corpora representing a particular area.

Finally, in the present study, the Laboratory Report is defined as follows (Author):

Undergraduate discursive genre whose communicative purpose is to present observed phenomena and interpret them in controlled conditions. Its predominant discursive organization is descriptive. Semiotically, it is configured preferably by the verbal, graphic, and mathematical modes. The relationship between participants is undergraduate student-writer-expert reader. The context of circulation is scientific. Its implied learning outcome is to analyze and apply procedures characteristic of the scientific method.

This characterization allows us to address the textual analysis considering a wide range of variables for genre identification; the learning outcome is particularly relevant, given the formative nature of this discursive genre.

### 2.2 Characterization of the technical report macro-genre in civil computer engineering

**Figure 1:**
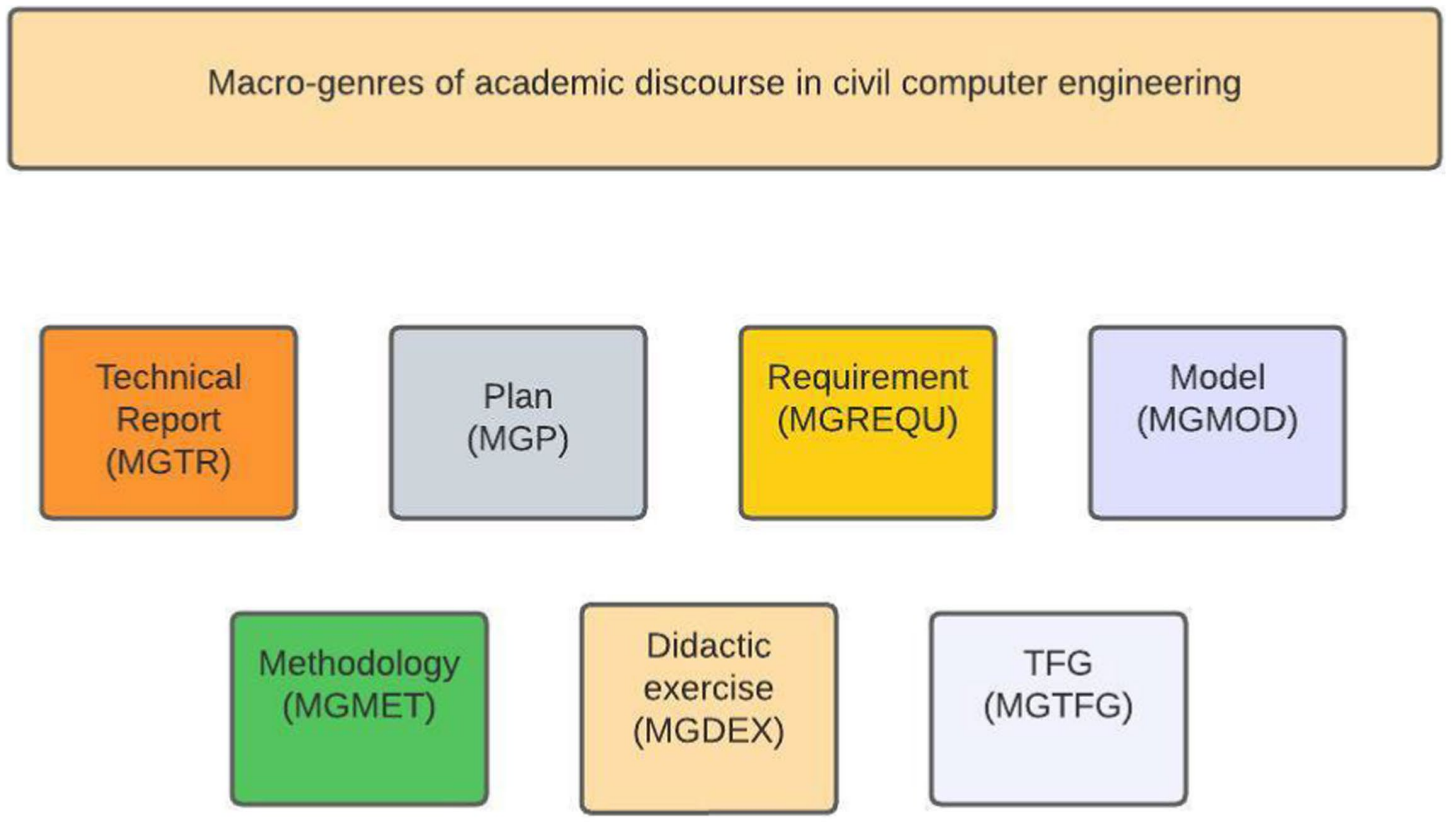
Macro-genres of academic discourse in civil computer engineering (MGEFIC).

The laboratory report is an academic training and epistemic genre of a mesogeneric nature which belongs to the Technical Report family or macro-genre. In fact, the curricular stage called *capstone plan* or terminal professional cycle articulates a great variety of genres (Devitt, 1991; Bazerman, 2004) that undergraduate students have to use according to their role in the community of practice. In this vein, the findings available from a previous study (Author) configure a map or genre system made up of thirty-three generic instances grouped in seven macro-genres or families. Figure 1 presents the macro-genres found:

This wide diversity is supported by the available literature on engineering writing in other higher education systems such as the British one. Nesi and Gardner’s (2006) early studies and Gardner (2008) have pointed out written production in engineering is relatively demanding in terms of genre variation. Through corpus-based research and ethnographic methods carried out in disciplinary departments, the authors conclude that undergraduate engineering students produce writing tasks belonging to the thirteen genre families, which range from essays to design specifications (including exercises, explanations, research reports, reviews, proposals, among others).

The latter reflects the multidisciplinary nature of engineering “that draws on disciplines from mathematics to management” (Gardner, 2016, p. 5). In addition, research on engineering vocabulary has demonstrated that significant differences exist between the vocabulary used by groups depending on cohort and academic cycle, and even between undergraduate and graduate students (Durrant, 2016). Durrant (2014) asserts that the vocabulary used in fourth year of engineering compared with that of third year is closest to business-related vocabulary. This can be explained by the steady increase in the importance given to project management in engineering undergraduate professional cycles as well as master programs. Moreover, the available research on the report genre in an undergraduate context confirms a much greater frequency of report writing in the last years of engineering studies (Gardner and Holmes, 2009; Nesi and Gardner, 2012; Parkinson, 2013).

Based on the analysis of the HÉLICE-2017 Corpus (Sologuren, 2020), the technical report macro-genre in computer civil engineering has been defined as follows:

The genres belonging to this category share the macro-purpose of stating the state of a procedure, experimental work, state of development, or a project status. The predominant sociosemiotic process of this macro-genre is to present.

Thus, the technical report is a macro-genre exhibiting a high genre variation, and it fulfils sociodiscursive roles within a community of practice and formative contexts:

These types of genres have as their central purpose to give an account of the state of progress of experimental work. It requires students to provide information from one or more sources, analyze such information and provide recommendations about the process developed based on the analysis (Author).

In fact, within this macro-genre, training genres converge which share writing practices aimed at (1) familiarizing students with the disciplinary concepts and methods—that is, these training genres are more of a *Pedagogical Text* (Gotti, 2014), (2) other practices focused on research writing, and (3) practices for a greater level of professionalization that prepares students for the workplace.

**Figure 2:**
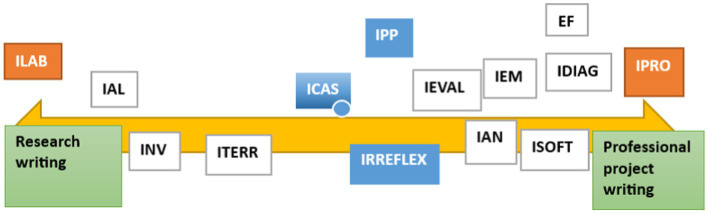
Genre continuum in the Technical Report macro-genre (MGITEC) in Civil Computer Engineering.

In this continuum represented in Figure 2, we can observe the emergence of networks of training genres or hybrid-type epistemic genres. A genre network in the terms proposed by Swales (2004) seeks to capture some notion of the current general framework or the global view more dynamically than does Bazerman’s construct of genre system. Genre networks refer to broader intertextual relations between genres in a specific discipline and in a specific learning community. Ultimately, they capture genre relations, which are always dynamic and constantly evolving.

Figure 2 illustrates a genre network which is heavily academic or research-oriented. It comprises four genres approaching a completely academic pole: *Lab Report (ILAB), Algorithm Report (IAL), Research Report (INV), and Field Report (ITERR)*. However, these exhibit hybrid features characteristic of a textual space (Bhatia, 2010) that is highly dynamic due to the convergence of different disciplines, specific writing practices, and academic and professional cultures: “Expert professional writers who constantly operate within and across generic boundaries, creating new but essentially related and/or hybrid (both mixed and embedded) forms to express their ‘private intentions’ within socially accepted communicative practices and shared generic norms” (Bhatia, 2015, p. 23).

At the other end of the continuum there is a numerous network of non-academic, professional-oriented genres that have been updated to pedagogically-oriented versions to develop a several types of skills expected in the workplace. These seven genres are the following: *Project Report* (*IPRO,* see Sologuren and Venegas, 2021), *Software Report (ISOFT)*, *Assessment Report (IEVAL)*, *Diagnostic Report (IDIAG)*, *Consulting Report (ICON)*, *Market Research Report (IEM)*, *Business Analysis Report (IAN)*, and *Financial Status Report (EF)*. They align not only with the applied engineering curriculum, but also with management education, finance, economy, and project management. In fact, the educational ends of these courses are in line with the following feature of the science-based engineer who graduates, recognized by Washington’s agreement:

WA11: Demonstrate knowledge and understanding of engineering management principles and economic decision-making and apply these to one’s own work as a member and leader in a team, to manage projects and in multi-disciplinary environments (International Engineering Alliance, 2014, p. 15).

In each of these textual spaces or constellations (Swales, 1990) which circulate at a specific time and educational cycle (terminal), the different members of the technical report macro-genre establish various types of embedding, mixing, and connection relations, whose understanding and practice allows the appropriation of the generic resources, in Bhatia’s terms (2015: 24): “Appropriation of generic resources is also common in various forms of hybrids, such as mixing, embedding and bending of genres.” Thus, an undergraduate engineer as a legitimate peripheral participant (Lave and Wenger, 2001) of a community of practice will have to become aware that genres do not operate in an isolated manner and that their acquisition during the learning trajectory is vital to be able to move toward positions of greater time and effort commitment within the communities of practice, and thus, to positions that build one’s professional identity (Noceti and Benedetti, 2010).

Moreover, at the center of the MGITEC continuum there are three heavily-curricular genres, that is, they target “goal driven classroom activities, devoted to the accomplishment of significant educational ends” (Christie, 2002, p. 22). These are genres whose possible expert versions in the academic and professional communities are less clear; these are generic resources characteristic of institutional contexts, and they adhere to classroom discourse (Cotos and Chung, 2019) from a wide perspective.

In this manner, the case report (ICAS), the professional internship report (IPP), and the reflective report (IRREFLEX) become useful preparation for the workplace, but their focus is the application of key disciplinary concepts, work methods, and the reflection arising from real cases or professional situations. ICAS, on the other hand, can be even more hybrid: if it is part of a business analysis, it can be integrated into the genre of business analysis report (IAN), whose target is a professional audience and whose aim is to persuade the reader to make a financial decision (Yeung, 2007).

Based on these results, there are three generic subcolonies or subfamilies: (1) *research*, (2) *curricular*, and (3) *professional* each represented by the most prototypical, relevant, and frequent genre in the students’ corpus. These genres, which act as nuclei in each of their networks, have been categorized within the technical report macro-genre as mesolevel type, as they share rhetorical and discursive characteristics with the members of their constellations as well as similar communicative purposes. These mesogenres allow the development of a research design, implementation, and appreciation (ILAB), the understanding of professional internships (ICAS), and the organization of an action plan (IPRO). All of this makes it possible to understand the production context of these academic training genres and their contextual relevance for the teaching of academic writing in a specific disciplinary area.

This article centers on the Laboratory Report mesogenre (ILAB). The following sections delve into this mesogenre within MGITEC by describing its rhetorical organization and its main discursive characteristics through the development of a rhetorical-functional model.

## 3 Methodology and corpus

This study focuses on academic writing within an engineering community of practice, an area which has not been sufficiently researched. The present research is qualitative and descriptive, based on genre’s empirical analysis. The methodological proposal is based on previous studies of the same research team on other genres in the Technical Report family (Author).

The subcorpus used to develop the ILAB genre’s rhetorical-functional model is made up of 112 written texts with an average word count of 2,483, and a total of 301,078 words. All the texts come from the multigenre corpus HÉLICE-2017. This ecological collection was built thanks to the contributions of 103 civil computer engineering students in their terminal undergraduate cycle, belonging to three Chilean universities of national and international prestige: Pontificia Universidad Católica de Valparaíso, Universidad Técnica Federico Santa María, and Universidad de Chile. The courses that promote the production of this genre are presented in the following section. This allows a curricular contextualization for the findings.

The texts making up the corpus are current (2015–2019), complete texts, writing tasks with a high or acceptable grade (higher than or equal to 5,5 on a scale of 7,0 as the highest grade), and part of a course plan within the terminal or professional cycle study plan. All of these characteristics make us consider them as validated by a community of experts. It should be noted that each of the voluntary participants contributing with their texts signed the corresponding informed consent that ensures the ethical treatment of the data.

For this study, we used a deductive-inductive model analysis to identify the moves and rhetorical steps making up the rhetorical-discursive organization of the identified genre (Biber et al., 2007). Move analysis was carried out through manual labeling (Upton and Connor, 2001; Wu et al., 2006; Ding, 2007; Bianchi, 2008). By applying the analytical steps, we achieved a description of the corpus-based discourse structure (Kanoksilapatham, 2007, 2011, 2015). Table 1 synthesizes the main methodological aspects for the genre analysis which were considered within a rhetorical discourse analysis (Manrique-Losada et al., 2019):

**Table 1:** Methodological phases for genre analysis (Manrique-Losada et al., 2019).

(i) Determine the rhetorical purpose of each genre.
(ii) Determine the function of each textual segment in its context.
(iii) Group the semantic-functional themes (steps).
(iv) Piloting the analysis in order to refine the code scheme.
(v) Develop a move and step protocol.
(vi) Code the complete set of remaining texts making up the subcorpus.
(vii) Carry out an inter-analyst validation.
(viii) Check the resulting code protocol.

Textual analysis also implies a quantitative description of the corpus’ rhetorical moves and steps. A move is conceptualized as a rhetorical unit carrying out a communicative purpose in a specific discursive genre, while a rhetorical step refers to a smaller rhetorical unit that allows the move to achieve its purpose (Swales, 1990, 2004). Rhetorical organization, on the other hand, is defined as “a genre’s functional structure by systematizing its rhetorical-discursive units and subunits” (Burdiles, 2015, p. 190).

A macromove is understood as a larger rhetorical unit than the move, and it enables rhetorical-functional analyses in longer, unstudied texts through is unitarian shape, as in the case of the macro-genre Final Degree Report (MGTFG) (Venegas, 2010) and the thesis genre (Parodi, 2008).

This is also observed in the Technical Report macro-genre (MGITEC) which is part of our HÉLICE-2017 student corpus. It is a functional-discursive unit that exhibits a communicative macropurpose and that structurally aligns with the larger sections of a macro-genre or a specific genre (introduction, theoretical framework, results, conclusion, etc.). In this vein, for the concept of rhetorical move and for the complete genre analysis, a key notion is the communicative purpose that the members of a discursive community will aim to fulfil (Meza and Moyano, 2023).

For the segmentation and assignment of communicative purposes, some of the criteria considered are the researcher’s previous knowledge, the institution’s material, the existing descriptions of some genres in other languages, the empirical information coming from the observation of several microcorpora, and expert judgments. The *QSR Nvivo pro 12 software* was used for coding, which allowed the characterization and validation of the rhetorical units by the principal investigator and two annotators trained for this purpose. Table 2 details the methodological steps during data analysis:

**Table 2:** Methodological steps for data analysis.

1. Review of the existing literature to look into descriptions of some of the genres found in other languages.
2. Configuration of each genre’s preliminary rhetorical-functional models with a microcorpus (33%).
3. Validation of the rhetorical-functional models with 3 engineering disciplinary experts and 2 experts on genre analysis.
4. Adjustments to the preliminary rhetorical-functional models, after the validation process.
5. Analysis of the full corpus (remaining 67%) based on the validated models.
6. Establishment of move occurrences in the corpus by distinguishing between obligatory (100-80%), very frequent conventional (79-60%), frequent usual (59%-40), infrequent (39-20%), exceptional (19-1%) in the corpus.
7. Identification of the distinctive textual characteristics.

We apply a bottom-up model that is complemented by the following specific methodological steps making up the analysis plan of this rhetorical-discursive organization (Burdiles, 2011, 2015; Manrique-Losada et al., 2019). After applying all the phases, we obtain a preliminary model of the rhetorical organization within the MGITEC family (Table 3):

**Table 3:** Analysis plan for the rhetorical-discursive characterization.

1. Random selection of four samples belonging to one of the most frequent genres in the subcorpus of the technical report macrogenre MGITEC: Laboratory Report (ILAB), making sure each sample belongs to a different university, within the three universities considered in this study, so as to be balanced.
2. Incremental construction of the preliminary model based on the manual analysis of the structure and superstructure in order to identify common rhetorical organization units.
3. Definition of the rhetorical moves as genre functional sections (Swales, 1990, 2004). At this stage, we consider the concept of macromove (Parodi, 2008) defined above, which considers a particular communicative purpose.
4. Identification of the purposes of a high hierarchy level or macropurpose compared with a set of smaller communicative purposes that enable the achievement of this general purpose, which are carried out through specific rhetorical moves and detailed rhetorical steps. We used as initial guides for analysis the proposals of Swales (1990, 2004); Yang and Allison (2003); Lorés (2004); Kanoksilapatham (2007), and Parkinson (2017); all of them are proposals for the analysis of scientific research articles, except the last study which focuses on the laboratory report.
5. Application of the rhetorical discursive mesomodel to the whole subcorpus considered.
6. Validation of the moves and steps protocol through the quantitative description of their degree of occurrence. The occurrence percentage of moves are described in the general methodology section.
7. Corroboration of the rhetorical-discursive model with an expert discipline informant: in order to check and validate the rhetorical-discursive analysis, we interviewed a lecturer in the area of civil computer engineering and an expert in genre analysis.

For data processing, we used text editing tools (Word) and spreadsheets (Excel) for data systematization and occurrences identification.

## 4 Results

The following sections present the resulting rhetorical-discursive model for the ILAB mesogenre. We also analyze and exemplify the ILAB’s rhetorical organization and provide a discussion of the findings, which focuses on the relations established by the different genres making up the MGITEC genre.

Writing a laboratory report not only enables the assessment laboratory work in engineering and science, but it also becomes a suitable choice to communicate the findings of empirical studies (Parkinson, 2017) and situate students in their own scientific activity (Dreyfus et al., 2016).

The number of courses that require the writing of this genre in this formative cycle continues being considerable; therefore, this activity is not exclusive of the first years of the major, but it remains throughout the study plan. Table 4 shows the courses that promote this genre’s update, which belong mostly to semester seven through ten:

**Table 4:** Civil computer engineering courses requiring ILAB.

Algorithm design and analysis	Computer networks
Computer systems workshop	Computational statistics
Scientific computing	Operations research
Introduction to data mining	Computational intelligence
Computational intelligence and robotics laboratory	Information and communication technologies
Information and communication technologies laboratory	

Gardner (2012), based on data from the *BAWE* corpus, points out that the laboratory report gives greater importance to the methodology and results sections, and that, although it is a canonical *Introduction-Methods-Results-Discussion (IMRD)* structure, it is different from published academic texts. In this sense, it is important to analyze the training genres on their own merit, taking into account the social context of knowledge evaluation and demonstration.

For the disciplinary community studied, the laboratory report can account for two types of experiences: one of simulation, which implies using computer tools for system design and analysis (Markvart and Castañer, 2003; Silvestre et al., 2008); and a practical one, which involves manipulating instruments and concrete artifacts such as circuits, parts, and components of different devices. In both cases, this laboratory activity is crucial for the development of knowledge, skills, and empirical thinking modalities in engineering and sciences (Parkinson, 2017). What follows presents and exemplifies the resulting rhetorical-discursive mesomodel for *ILAB*.

The rhetorical organization of *ILAB* is made up of four macromoves, twelve moves, and seventeen steps. Each of these rhetorical units and microunits are specified in Table 5 (presented later), which details the rhetorical discursive mesomodel, as well as Table 6, which describes the identified steps, many of which are recursive or cyclical as we shall see in subsequent paragraphs.

**Table 5:** Multimodal artifacts (Boudon and Parodi, 2014, p. 180).

Formula	Graph
Artifact built preferably from three modalities: mathematical, verbal, and typographical. The formula makes it possible to establish relations between mathematical properties or assign values in a numerical equation (180).	Artifacts that preferably combine these four modalities: verbal, graphic, mathematical, and typographical. In it, a visual summary of statistical information is represented as a picture (180).

**Table 6:** Global rhetorical organization of ILAB’s MM1.

Macromove 1’s rhetorical organization (MM1): Introduce the reader to the laboratory experience		
Move/Step	Communicative function	Example from the HÉLICE-2017 corpus
Move 1I: Establish the topic of the experiment/simulation		
Step 1.1: Indicating the importance	Stating the importance of the topic	a. From biology to astronomy, these systems have significantly contributed to the respective studies, leading to unprecedented advances (421-1).^1^ b. Technology has acquired an across- disciplines role, fostering the quality of learning and the development of skills in society, increasing economic productivity, among others (419-3).
Step 1.2: Presenting the known information	Provide general background information	c. Image processing applications extend to multiple areas (ICI_438-2). d. that allows faster search and insertion operations in very large datasets (DCC_75-3).
Step 1.3: Outlining the structure	Present the report’s organization	e. For this, we will explain the structure of an R-tree, how the insertion and search processes work, and how overflows are handled with the different algorithms (DCC_75-3). f. The most relevant results of the training, validation, and testing process will be presented, along with the subsequent analyses and conclusions (420-5).
Move 2I: Advance a hypothesis		
Move 3I: Introduce the experiment		
Step 3.1: Establishing the purpose	Indicate the aim of the experiment	g. The aim of this Laboratory Activity is to implement and analyze simple algorithms of movement detection and object tracking (438-2). h. This report’s aim is to present in detail task 1’s resolution process, which consists in creating and comparing two algorithms (or heuristics) used in an R-tree construction (DCC_75-3).

^1^The first number identifies the document in the student corpus, and the second number corresponds to the page from where the textual evidence is extracted.

**Figure 3:**
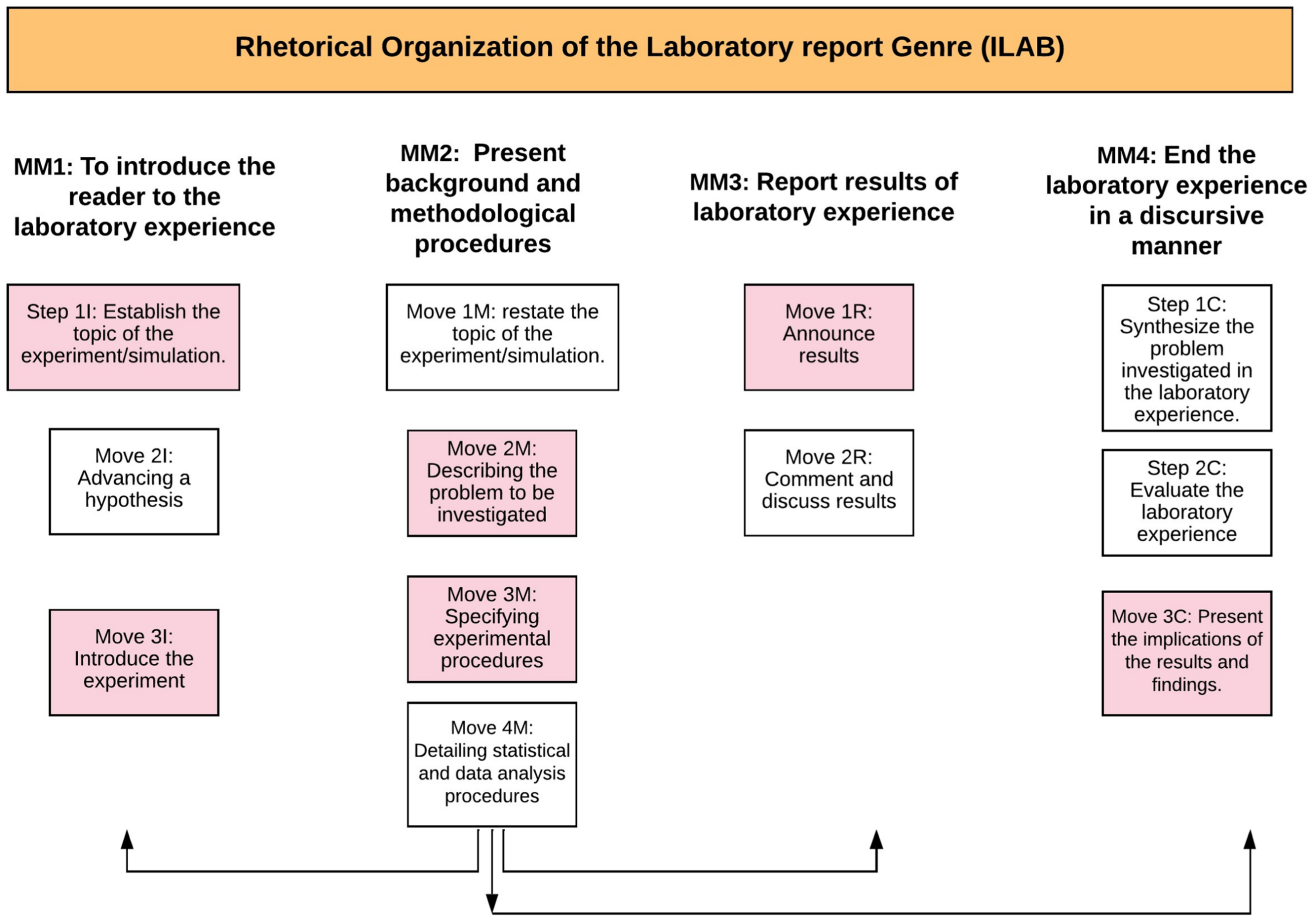
Rhetorical discursive mesomodel of the Laboratory Report (ILAB).

Now we will outline the resulting functional organization of this Mesogenre^1^ which exhibits an *I-M-R-C* rhetorical sequence: *Introduction-Methods-Results and Conclusion*, combining a sequential display of rhetorical moves with iterations *from* and *to* methodology’s rhetorical macromove 2 (MM2), given the centrality of this section in the analyzed texts. In this manner, we can assert that this MM2 constitutes the nuclear macromove (Figure 3):

In fact, the methodology’s rhetorical macromove (MM2), “*Presenting background and methodological procedures*” are highly dynamic, due to the recursiveness of its rhetorical steps and its functional flexibility. This cyclic property was considered in the reformulation of Swales’ *CARS* model (2004), and it means a move or step can occur again in other text sections or macromoves, as they are conceptualized in this research.

This dynamic nature is relevant for laboratory reports, as this section or macromove in the scientific discourse works as an epicenter: “It is an explicit bridge between the review of relevant literature and the newly-obtained results” (Cotos et al., 2017, p. 92). This epicentric character accounts for the emphasis the discipline gives to methodology, which aligns with what professors and students express in Chapter 7 in relation to the writing of the MET microgenre.

Another element that is worth pointing out relates to the use of multimodal artifacts to achieve the communicative purposes of this academic training genre. In fact, as demonstrated by Castro-Alonso and Fiorella (2019), visual–spatial processing is very important not only for the area of health sciences (such as medicine, anatomy, surgery, dentistry), but also for the natural sciences (such as biology, chemistry, physic, geology, meteorology), which are present in the curricula and the problems of applied engineering. Moreover, the role played by multimodal artifacts has been highlighted in economics discourse (Parodi and Julio, 2017).

The multimodal artifacts found in the ILAB subcorpus are the formula and the graph. For the academic discourse of economics, Boudon and Parodi (2014) define these artifacts as follows:

Thus, based on these starting-point definitions, new research cycles on the student writing corpus collected for this project should look into the function of these rhetorical devices in different text display patterns, for example, in the problem-solution types (Hoey, 1983), which are frequent in the engineering academic discourse. Based on the analysis of the 112 texts making up the subcorpus, Table 7 shows the frequency of rhetorical moves and steps:

**Table 7:** Moves and steps of the ILAB Mesogenre.

IMRC moves and steps	Frequency
**Move 1I: establish the topic of the experiment/simulation**	**96**
Step 1.1: Establishing the importance	66
Step 1.2: Presenting the known information	100
Step 1.3: Outlining the structure	85
**Move 2I: Advance a hypothesis**	**61**
**Move 3I: Introduce the experiment**	**100**
Step 3.1: Establishing the purpose	72
**Move 1M: Restate the topic of the experiment/simulation**	**73**
Step 1.1: Presenting the known information	100
**Move 2M: Describe the inquiry problem**	**100**
Step 2.1: Establishing the relation with the specialized literature	84
Step 2.2: Presenting the background to address the procedure	100
**Move 3M: Specify experimental procedures**	**93**
Step 3.1: Listing the materials	67
Step 3.2: Detail of procedures	100
Step 3.3: Visualization of procedure through a diagram or formula	76
**Move 4M: Detail statistical and data analysis procedures**	**69**
**Move 1R: Announce results**	**100**
Step 1.1: Justification of the methodology	36
Step 1.2: Focusing results	100
Step 1.3: Display/visualization of results through figures, tables, and graphs	100
Step 1.4: Calculation of results and/or development of equations	87
**Move 2R: Comment and discuss results**	**74**
Step 2.1: Explanation of results	100
**Move 1C: Synthetize the inquiry problem in the laboratory experience**	**77**
**Move 2C: Evaluate the laboratory experience**	**73**
**Move 3C: Present the implications of results and findings**	**80**
Step 3.1: Establishing limitations	55
Step 3.2: Indicating suggestion for improvement	43

As we can see, six of the twelve moves are obligatory (80% or more) and the remaining six moves are very frequent conventional (between 60 and 79%) or frequent usual (40% and more). Regarding steps, ten out seventeen are obligatory, four are frequent conventional, one frequent usual, and two infrequent. Tables 6, 8 show the communicative functions determined for the moves and steps of each ILAB genre’s macromove, based on the HÉLICE-2017 corpus analysis. It is therefore a model of communicative purposes. Moreover, each rhetorical step example shows the lexicogrammatical clues that allow the manual corpus annotations.

**Table 8:** Global rhetorical organization of ILAB’s MM2.

Macromove 2’s rhetorical organization (MM2): Presenting background and methodological procedures		
Move/Step	Communicative function	Example from the HÉLICE-2017 corpus
Move 1M: Restate the topic of the experiment/simulation		
Step 1.1: Presenting known information	Provide information available in the specialized literature	i. The frame difference motion detection algorithm is one of the simplest detection methods (438-3). j. An artificial neural network is defined as a “linear mapping system, whose structure is based on principles observed in human and animal nervous systems.” (419-5).
Move 2M: Describe the inquiry problem		
Step 2.1: Establishing the relation with the specialized literature	Link the experience with the specialized bibliography	k. The concept of linear separability of sets can be seen in Illustration 2 (420). l. Gradient descent algorithm [2] The present method used in neural network training defines a function that is given by the system error, which depends on the synaptic weights that make it up (421).
Step 2.2: Presenting the background information to address the procedure	Present preparatory information needed to carry out the experiment	m. Now, it should be noted that the weights variations in each training adjustment is represented by the learning rate 𝛼, which, if it is too small, it will imply the algorithm’s low convergence speed. In contrast, if it is large, oscillatory effects will appear in the convergence (420-10). n. The general structure of the convolutional network for the present experience is the following (421).
Move 3M: Specify experimental procedures		
Step 3.1: Listing the materials	Listing the necessary materials	ñ. Cisco Catalyst 3,650: • Switching capacity: 88 Gbps • Stack bandwidth: 160 Gbps • Total number of MAC addresses: 32000 • Total number of IPv4 routes: 24000 • DRAM: 4 Gb • Flash: 2 Gb • Total switched virtual interfaces (SVIs): 1000 • Total routed ports per 3,650 stack: 208 • Number of access points per switch/stack: 25 • Number of wireless clients per switch/stack: 1000 (206-3) o. HP 3800-24G-2SFP+: • Processor: HP ProVision ASIC/ARM @ 350 MHz • Flash: 4 GB • SDRAM: 2 GB • Packet buffer size: 18 MB dynamic • Latency 1,000 Mb: <2.8 microseconds (64 Bytes packs) • Latency 10 Gb/s: <1.9 microsegundos (64 Bytes packs) • Performance: over 65.4 million packs per second (64 Bytes packs) • Switching capacity: 88 Gb/s • IPv4 Routing table size: 10000 • MAC address table size: 65500 (207-4).
Step 3.2: Detailing the procedure	Present the procedures needed for the experiment.	p. In this algorithm, a background model is developed by calculating the mean and standard deviation of an image set for the same pixel, so the algorithm learns to recognize the scene’s background (438-7). q. When the optimization process of a neural network’s costs function deviates from the established criteria, it must be penalized so as to correct the algorithm, in the weight update (420-9).
Step 3.3: Visualizing the procedure through diagram or formula	Graphically Project methods and procedures implied in the experiment or simulation	r. 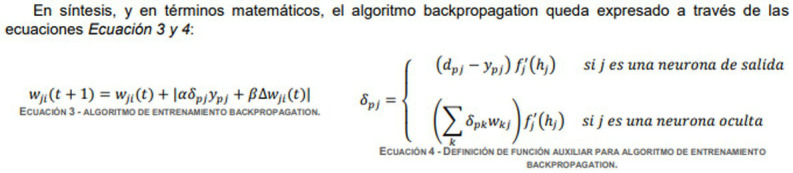 (420-9). s. 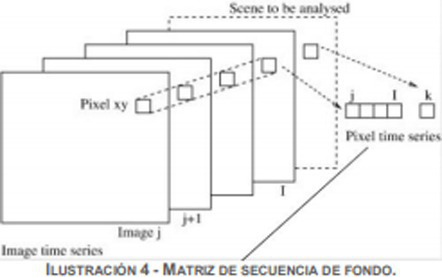 (438-7).
Move 4M: Detailing statistical and data analysis procedures		

Move 3I, which is obligatory, oscillates between stating the objective from the point of view of a researcher writer or from the point of view of the student’s learning activity (examples *a* and *b*). This illustrates the social formative (training) purpose of this genre and the organization texts adopt to situate the experimental work of the civil computer engineering student (Tables 9, 10).

**Table 9:** Global rhetorical organization of ILAB’s MM3.

Macromove 3’s rhetorical organization (MM3): Report results of the laboratory experience		
Move/Step	Communicative function	Example from the HÉLICE-2017 corpus
Move 1R: Announce results		
Step 1.1: Justifying the methodology	Explain methodological decisions	t. The inclusion of the bias gradient per layer, weight gradient per layer, evolution accuracy, and costs evolution graphs will allow the analysis of the effects of the variations carried out (421-8). u. The detection of object dynamics in the frames through the present algorithm presents greater robustness facing external noise, although the difference criterion is used with an associated threshold (438-7).
Step 1.2: Focusing results	Highlight obtained results	v. As it can be observed in Results 7, the average Matrix of the sequence of background images is able to establish a robust frame of reference (438-10). w. As part of the obtained results, Figure 1 shows that as the number of inserted elements increases, the difference in insertion times (ms) applying LinearSplit and QuadraticSplit, also increases (75-15).
Step 1.3: Display/visualization of results through figures, tables, and graphs	Represent experiment findings	x. 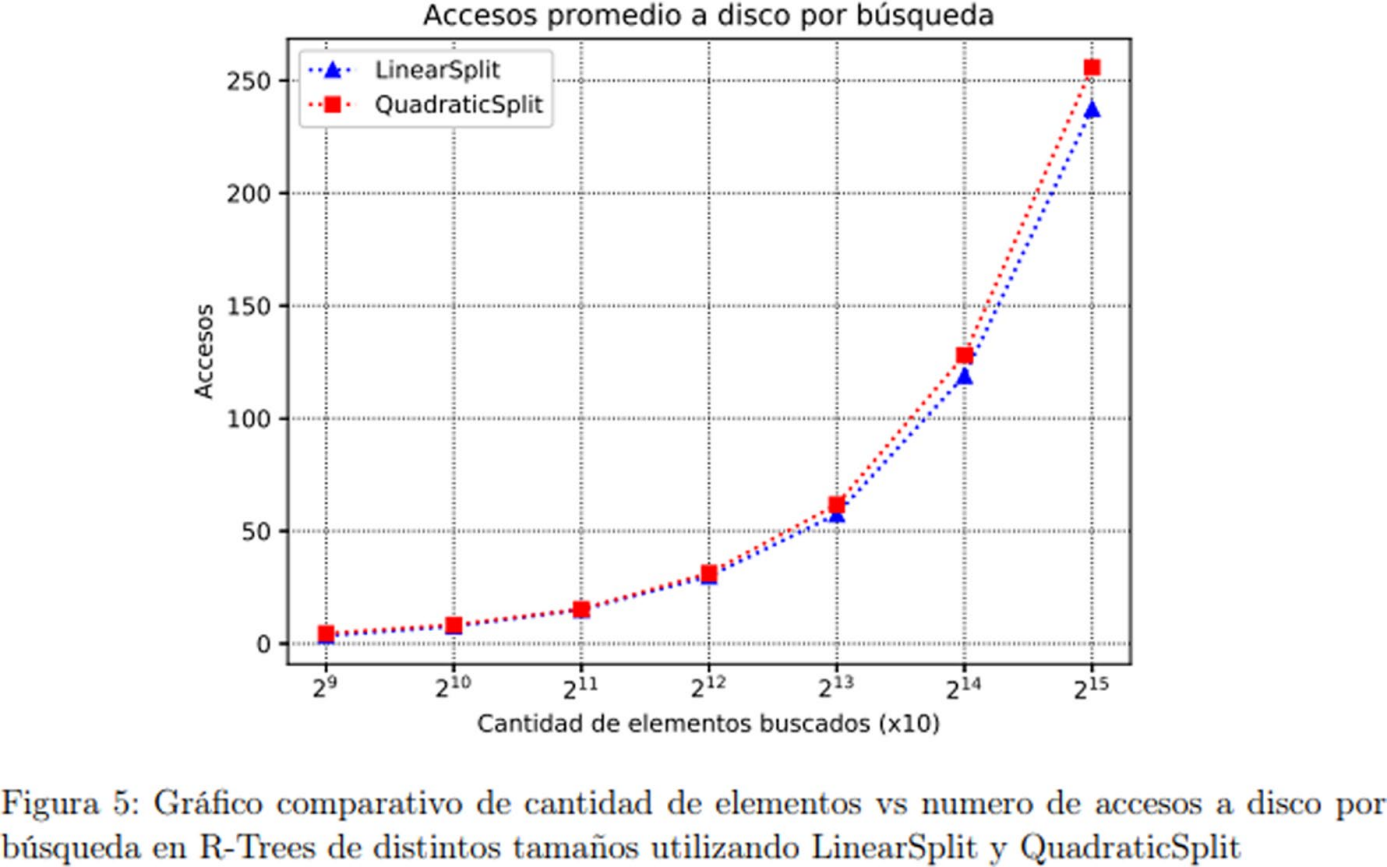 (75-14) y. 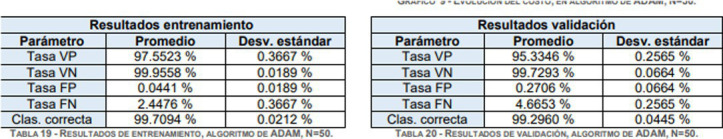 (420-14).
Step 1.4: Calculating results and/or presenting equations	Calculate to obtain quantitative data	z. the optimal number of units is established in the hidden layer, which makes it possible to obtain the maximum average percentage of classifications, 50. This is supported by Función 4 (420-16). aa. 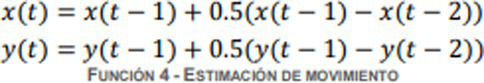 (438-13).
Move 2R: Comment and discuss results		
Step 2.1: Explaining results	Provide a coherent explanation of the obtained results	bb. This is because the neural network’s optimization process deviates from the established criteria, so the weight updates are penalized and the number of epochs increases (420-17). cc. Based on the experiences carried out, we are able to determine that a convolutional neural network classifies with a greater accuracy and lower associated costs than one with a multilayer perceptron, both in the training and classification periods (421-15).

**Table 10:** Global rhetorical organization of ILAB’s MM4.

Rhetorical organization of macromove 4 (MM4): End the laboratory experience in a discursive manner		
Move/step	Communicative function	Example from the HÉLICE-2017 corpus
Move 1C: Synthesizing the inquiry problem in the laboratory experience		
Move 2C: Evaluate the laboratory experience		
Move 3C: Present the implications of results and findings		
Step 3.1: Establishing limitations	Identify the study’s limitations	dd. Even when these uniformity conditions are not always met, the delivered estimations are adequate, taking into consideration the simplicity of the implemented methods (438-15). ee. However, it has some disadvantages that ranked them below dynamic routes. • They are not easy to implement in a large network. • Handling the static configurations can become tedious when the network begins to grow. • If a link fails, static routing cannot redirect traffic (206-19).
Step 3.2: Indicating suggestions for improvement	Identify improvements that can be applied	ff. Other points to take into account when choosing RIP over a static routing is route search speed and the handling of redundancy (broken links and redirection (206-19)]. gg. it is important that each of the components of the robotic system is in good condition, since with them the processes of data acquisition, processing, and decision-making will be carried out (434-10).

## 5 Discussions and conclusion

The rhetorical-discursive model presented shows a hybrid form of the laboratory report genre in civil computer engineering education. In fact, the analysis of its rhetorical organization evidences both its curricular writing and its research writing nature. An example of the latter is the display of macromove 3: “*Report results of the laboratory experience*.” In this vein, it is a genre of the Technical Report family which is very close to the academic forms of communication, as it can be seen in the rhetorical organization of macromove 2: “*Present background and methodological procedures*.” Thus, MM2’s rhetorical steps aim at the development of research and interpretation tools: description, justification, definition, visualization, and explanation; in contrast, MM3’s steps aim at the development of application, synthesis, and integration in academic contexts. It implies, therefore, the design, implementation, and appreciation of simulated or physical experiences that make it possible to contrast hypotheses about relevant scientific phenomena.

The laboratory report genre thus becomes an implementation genre fulfilling a role of polar nucleus within the Technical Report family or macro-genre. This is because it consists of a composition task that combines elements that are typical of research writing with others typical of curricular writing, and in so doing, it develops diverse higher-order cognitive skills in engineering students. This mixture and polarity within the continuum makes it a relevant discursive practice in engineering education, which can be developed in depth and also assessed in engineering classrooms. In this way, students will be able to apply strategies to adapt writing to a more investigative or applied context and transfer their rhetorical knowledge in the production of new specialized genres. Table 11 shows this hybrid nature and educational potential:

**Table 11:** Rhetorical step that states the writing purposes of laboratory reports.

ILAB
Step 3.1: Establishing the purpose
g. The objective of this Laboratory Activity is implementing and analyzing simple algorithms of movement detection and object tracking (438-2).

**Figure 4:**
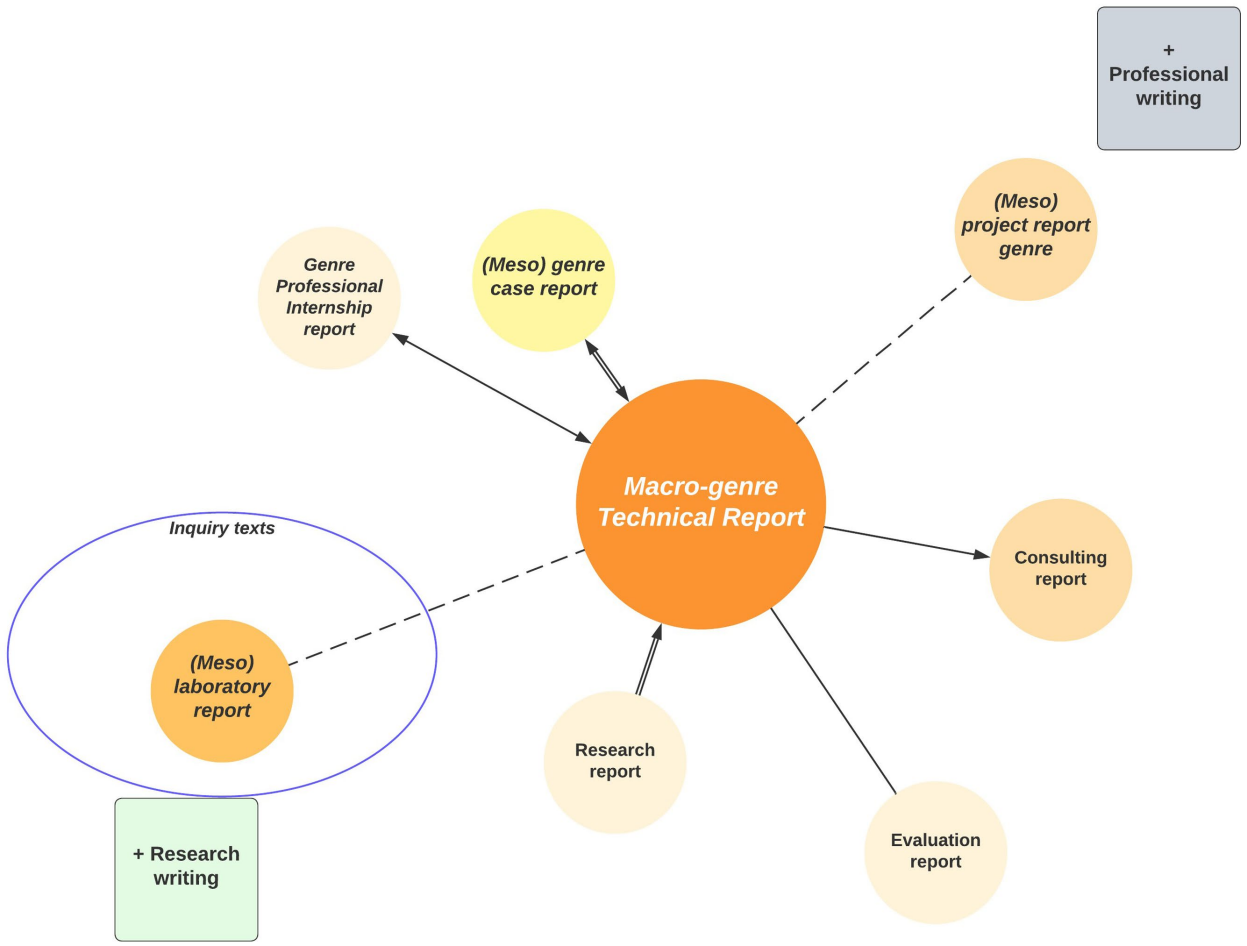
Network and continuum of macro-genre technical report in civil computer engineering.

ILAB is then situated in the research writing pole, and in this sense, it aligns more prototypically with the curricular activities of the engineering classroom. Figure 4 shows the location of ILAB within the macrogeneric family as a mesogenre oriented toward developing experimental research:

In fact, this mesogenre defines and projects to a great extent the discursive performance of students in this cycle as advanced writers: “I would like to call it ‘discursive performance’, which extends the scope of analysis from genres as discursive product to professional practice that all discursive acts tend to accomplish” (Bhatia, 2016b, pp. 21–22). Thus, from an area of the discipline’s nucleus, the circulation context is clearly scientific, but at the same time it is an evaluation academic genre (Jarpa, 2016) that establishes different discursive trajectories with other genres in the university classroom. For example, with the “scientific research article” that reports the results of an experimental inquiry or with “methodological accounts” that propose experimental designs to address various problems around study.

In the same manner, these discursive trajectories (Sabaj, 2017) are displayed during the writing process in different stages and parts of a genre (Breeze, 2016) as it can be seen in the corroboration interview carried out with the program’s lecturers:

“We do have the concept of *writing reports* when we have to submit something. That is very present, even from the beginning with basic sciences courses. In all the physics laboratories, for example, the student has to write a *pre-report* and a *post-report* after [the laboratory] experience.” (P06_ICI_10-6)

“…and they iterate that *report from the beginning of the semester, and they have three iterations.* Then in the first one they do a smaller version, in the second one they improve that text and explore a *new part of the document,* and in the third one they improve all the previous work and submit a final part as result.” (P05_ICI_12-7)

Therefore, each genre becomes a phase in the genre chain (Swales, 2004) which will enable the fulfillment of the communicative macropurposes with a specific chronological order. It is a genre with considerable internal variability in terms of themes, argumentation and analysis procedures, as well as ways to address the issue; moreover, it shows various possible rhetorical move and step combinations that can be updated, since it is a genre with only six obligatory moves out of twelve, which are also distinctively cyclic (as can be seen in Figure 3). The latter makes it more flexible and adaptable for formative and academic needs, and it also makes the genre suitable for combination with other types of reports in the technical report family. In addition, the various multimodal resources such as graphs, formulas, and tables play a key role in this genre, to the extent that many times they become rhetorical steps or tactical cognitive strategies of the student-writer. Es decir, queda en evidencia que las funciones comunicativas de las unidades retóricas se pueden expresar no solamente vía recursos lingüísticos, sino que también a través de otros modos de construcción del significado como el tipográfico, matemático y visual. Los recursos multimodales son por tanto parte esencial del género.

However, the corpus analysis shows the tendency to emphasize the methodology and results sections of the practical experience or simulation. In fact, the rhetorical moves of the discussion and conclusion sections have a lower frequency than the central moves involving the presentation of results. The analyzed texts tend not to signal the closing generic stage. In this sense, we observe a tendency toward fragmentation in laboratory report writing, where the emphasis is put on the presentation of the experiment or virtual simulation results. This weakness in the creation of the discussion section is expressed by the professors as one of their students’ writing difficulties. Nevertheless, this concern does not lead to an action or concrete initiative to address this issue in the professors’ discourses. Thus, the development of critical thinking skills becomes an interesting future direction to inform the curricular innovation processes in undergraduate engineering.

Our research objective was to describe the rhetorical organization of this training genre in a key engineering subdiscipline. This research significantly contributed to the study of the rhetorical-discursive sphere in the Spanish language, and consequently, to the description of a discursive genre typical of an engineering disciplinary area. In so doing, the study contributes to complete the genre mapping of disciplinary discourses. Moreover, in terms of projections, the next step will be to relate the discursive rhetorical units with the preferred lexicogrammatical resources, contributing to the development of local grammars. Another important aspect is to promote the transfer of this new rhetorical knowledge to pedagogical devices and teach hybrid genres that combine curricular and reflective writing with the kind of writing expected in academic and professional settings, as it occurs in the laboratory report.

Finally, another projection of this research will be to systematically relate the teaching-learning methodologies of the civil computer engineering classroom, in which the production of texts (e.g., training genres produced and used as well as the associated writing tasks to produce those genres) is relevant for the achievement of relevant disciplinary learning outcomes. The in-depth study of this pedagogical-curricular triad is needed to understand the role of this genre in text comprehension and production processes, the cognitive demands it mobilizes, the writing purposes in academic and professional settings, and the degree of connection it has with tasks considered as of highest relevance in the professional career.

In this sense, these variables should be added to the study of texts in Spanish. Writing in the 21st century, as Montolío (2019) asserts, implies knowledge transfer, and the linguistic work— particularly of applied linguistics—has the challenge of linking its expert work to the challenges of current society. As discursive genre analysts, we should respond to the challenges implied in the formulation and resolution of problems linked to the comprehension and production of texts in relevant areas for current communities.

## Data availability statement

The raw data supporting the conclusions of this article will be made available by the author, without undue reservation.

## Ethics statement

The studies involving humans were approved by Comité ético-científico CEC Universidad de Los Andes, Chile (Scientific and Ethical Committee CEC Universidad de Los Andes, Chile). The studies were conducted in accordance with the local legislation and institutional requirements. The participants provided their written informed consent to participate in this study.

## Author contributions

ES: Conceptualization, Data curation, Formal analysis, Funding acquisition, Investigation, Methodology, Project administration, Resources, Supervision, Visualization, Writing – original draft, Writing – review & editing.

## Footnotes

^1^Obligatory rhetorical moves are highlighted in pink.
